# Tracking of enzymatic biomass deconstruction by fungal secretomes highlights markers of lignocellulose recalcitrance

**DOI:** 10.1186/s13068-019-1417-8

**Published:** 2019-04-01

**Authors:** Gabriel Paës, David Navarro, Yves Benoit, Senta Blanquet, Brigitte Chabbert, Bernard Chaussepied, Pedro M. Coutinho, Sylvie Durand, Igor V. Grigoriev, Mireille Haon, Laurent Heux, Charlène Launay, Antoine Margeot, Yoshiharu Nishiyama, Sana Raouche, Marie-Noëlle Rosso, Estelle Bonnin, Jean-Guy Berrin

**Affiliations:** 1 FARE Laboratory, INRA, Université de Reims Champagne-Ardenne, Reims, France; 2 INRA, Aix Marseille Univ., UMR1163, BBF, Biodiversité et Biotechnologie Fongiques, Marseille, France; 3 INRA, Aix-Marseille Univ., UMR1163, CIRM-CF, Marseille, France; 4 IFP Energies Nouvelles, Rueil-Malmaison, France; 5 CNRS, Aix-Marseille Univ., UMR7857 AFMB, Architecture et Fonction des Macromolécules Biologiques, Marseille, France; 6 INRA, UR1268 Biopolymères Interactions Assemblages, Nantes, France; 7 US Department of Energy Joint Genome Institute, Walnut Creek, CA USA; 8 Department of Plant and Microbial Biology, University of California Berkeley, Berkeley, CA USA; 9 CNRS, Univ. Grenoble Alpes, CERMAV, Grenoble, France

**Keywords:** Biomass, Filamentous fungi, Enzymatic degradation, Saccharification, Hydrolysis, Water sorption, Glucose release

## Abstract

**Background:**

Lignocellulose biomass is known as a recalcitrant material towards enzymatic hydrolysis, increasing the process cost in biorefinery. In nature, filamentous fungi naturally degrade lignocellulose, using an arsenal of hydrolytic and oxidative enzymes. Assessment of enzyme hydrolysis efficiency generally relies on the yield of glucose for a given biomass. To better understand the markers governing recalcitrance to enzymatic degradation, there is a need to enlarge the set of parameters followed during deconstruction.

**Results:**

Industrially-pretreated biomass feedstocks from wheat straw, miscanthus and poplar were sequentially hydrolysed following two steps. First, standard secretome from *Trichoderma reesei* was used to maximize cellulose hydrolysis, producing three recalcitrant lignin-enriched solid substrates. Then fungal secretomes from three basidiomycete saprotrophs (*Laetisaria arvalis, Artolenzites elegans* and *Trametes ljubarskyi*) displaying various hydrolytic and oxidative enzymatic profiles were applied to these recalcitrant substrates, and compared to the *T. reesei* secretome. As a result, most of the glucose was released after the first hydrolysis step. After the second hydrolysis step, half of the remaining glucose amount was released. Overall, glucose yield after the two sequential hydrolyses was more dependent on the biomass source than on the fungal secretomes enzymatic profile. Solid residues obtained after the two hydrolysis steps were characterized using complementary methodologies. Correlation analysis of several physico-chemical parameters showed that released glucose yield was negatively correlated with lignin content and cellulose crystallinity while positively correlated with xylose content and water sorption. Water sorption appears as a pivotal marker of the recalcitrance as it reflects chemical and structural properties of lignocellulosic biomass.

**Conclusions:**

Fungal secretomes applied to highly recalcitrant biomass samples can further extend the release of the remaining glucose. The glucose yield can be correlated to chemical and physical markers, which appear to be independent from the biomass type and secretome. Overall, correlations between these markers reveal how nano-scale properties (polymer content and organization) influence macro-scale properties (particle size and water sorption). Further systematic assessment of these markers during enzymatic degradation will foster the development of novel cocktails to unlock the degradation of lignocellulose biomass.

**Electronic supplementary material:**

The online version of this article (10.1186/s13068-019-1417-8) contains supplementary material, which is available to authorized users.

## Background

Lignocellulosic biomass (LB) is considered as a sustainable and alternative source of fuels, chemicals and materials. Valorisation of LB is expected to favour the transition from a fossil to a renewable carbon-based economy (so-called bioeconomy), thus limiting greenhouse gas emission and climate change, which have become strong policy priorities of the United Nations in the last years [1]. LB from grass and wood is mainly composed of three types of polymers that account for more than 90% of the plant dry weight: cellulose, hemicelluloses and lignin [2]. The high chemical and structural complexity of LB at different scales is a strong limitation for the development of economically viable processes [3]. To tackle the recalcitrance of LB [4, 5], a physico-chemical pretreatment step is mandatory to “open” the network of polymers, so that enzymatic catalysts can access and convert polysaccharides into oligosaccharides and monosaccharides. Enzyme action is often hampered by structural features, which limit or prevent progression of the enzymes towards their substrate [6–8] and by chemical motifs (such as hydrophobic clusters made by lignin), which are known to bind enzymes more or less irreversibly, making them unavailable for their substrate [9, 10]. In particular, lignin is an important factor [11] involved in non-specific interactions with enzymes [12]. As a result, the enzyme loading used in bioconversion of LB must be high enough to circumvent these limitations, impacting the cost of the enzymatic hydrolysis step considered as a bottleneck to the establishment of cost-competitive biorefineries [3, 13].

In order to optimize the saccharification step, mimicking strategies used by microorganisms that efficiently decay plant biomasses seems a relevant approach [14]. In nature, filamentous fungi are the most efficient decayers of LB with the secretion of a large array of complementary enzymes targeting the different components of plant cell walls [15]. To date, the enzymatic cocktails obtained from the industrial workhorse *Trichoderma reesei* are very efficient on cellulose [16, 17]. However, LB conversion is still not optimal due to the paucity of some key enzyme activities such as oxidative enzymes targeting the recalcitrant fraction of LB [18, 19]. Therefore, fungal biodiversity is seen as a promising source of lignocellulose-acting enzymes to improve *T. reesei* enzyme cocktails.

Regarding LB characteristics, recalcitrance to enzymatic hydrolysis is related to several chemical factors (lignin and relative monolignol contents [20]; hemicellulose acetyl groups content [21]; water retention properties [22]) and structural factors (cellulose accessibility, crystallinity and degree of polymerization, porosity [8]) (many factors reviewed in [7]). Several studies appear contradictory regarding the impact of these factors, limiting the emergence of universal trends, because (i) they often focus on specific plant species, whereas LB composition and structure is species-dependent; (ii) pretreatments are not optimized and often not industrially compatible; (iii) one factor possibly determining recalcitrance can be measured by various technical approaches (for example at least five analytical methods exist to measure cellulose accessibility [23]), hindering comparative analyses; (iv) enzymatic industrial cocktails have unknown composition, which does not help to figure out the importance of specific activities on recalcitrance [24].

The objective of this study was to assess the saccharification potential of LB by performing sequential hydrolysis by fungal secretomes, in order to understand the recalcitrance of residual products and to define saccharification recalcitrance factors, which can be quantified and therefore called markers. We have applied an industrially-type steam-explosion pretreatment [25] to substrates issued from an energy crop (miscanthus), a hardwood (poplar) and an agriculture by-product (wheat straw), which are representative LB feedstocks. Sequential action of the enzymatic cocktail of *T. reesei* and then of various fungal secretomes selected for their ability to efficiently degrade LB was followed [14, 26]. The characterization of the substrates and products along the hydrolysis steps was used to highlight chemical and structural LB markers correlated with saccharification, thus indicating recalcitrance level, independently from the substrates and from the fungal secretomes considered.

## Materials and methods

### Substrate pretreatment

Steam-exploded poplar (*Populus nigra* × *deltoides*), wheat straw (*Triticum aestivum*, Haussmann variety) and miscanthus (*Miscanthus* × *giganteus*) were used as raw substrates (R0 samples). The steam explosion process was conducted under the following conditions: miscanthus 190 °C, 7.5 min, 0.4% H_2_SO_4_; wheat straw 170 °C, 7.5 min, 0.35% H_2_SO_4_; poplar 195 °C, 7.5 min, 0.7% H_2_SO_4_, as previously described [25] (Additional file 1: Figure S1).

### Fungal strains

The strains *Artolenzites elegans* BRFM 1663 (subsequently named *A. elegans*), *Trametes ljubarskyi* BRFM 1659 (*T. ljubarskyi*) and *Laetisaria arvalis* BRFM 514 (*L. arvalis*) (deposited by “Université Joseph Fourier”, Grenoble, France, as CMPG 934) were obtained from the CIRM-CF collection (International Centre of Microbial Resources dedicated to Filamentous Fungi, INRA, Marseille, France). All strains were identified by morphological and molecular analysis of ITS (Internal Transcribed Spacer) sequences using the expert database Fungene-db [27] (http://www.fungene-db.org) or Genbank [28]. The strains were maintained on malt agar slants at 4 °C. The genome sequencing and assembly, and gene structural and functional annotations for *T. ljubarskyi* and *A. elegans* were performed at the Joint Genome Institute. All data are publicly available on the Mycocosm portal of these genomes (https://genome.jgi.doe.gov/Tralj1/Tralj1.home.html; https://genome.jgi.doe.gov/Artel1/Artel1.home.html). Global transcriptomic data of *L. arvalis* was obtained from Navarro et al. [26]. The *Trichoderma reesei* TR3002 strain (subsequently named *T. reesei*) was the cellulase hyperproducer strain CL847 containing a copy of an evolved variant of the *bgl*1 gene (strain TR3002) [29].

### Production of secretomes

*Trichoderma reesei* (strain TR3002) was grown at 27 °C in a 3 L bioreactor with a culture volume of 2 L. Fungal biomass was produced in a batch phase, on a mineral medium as described previously [30], containing 30 g L^−1^ glucose as a carbon source. Enzyme production was done in fed batch, feeding a 500 g kg^−1^ sugar solution composed of glucose/lactose/xylose at a 70:25:5 ratio (feeding rate of 2 mL h^−1^ during 220 h) to favour the secretion of both cellulases and hemicellulases. The culture broths (secretomes) were ultra-filtered (5 kDa cut-off membrane) and then stored at 4 °C until use.

Based on previous studies [14, 31], fungal cultures of basidiomycetes were performed in 250 mL baffled Erlenmeyer flasks with 100 mL medium containing 2.5 g L^−1^ of maltose as a starter, 1.842 g L^−1^ of diammonium tartrate as a nitrogen source, 0.5 g L^−1^ yeast extract, 0.2 g L^−1^ KH_2_PO_4_, 0.0132 g L^−1^ CaCl_2_/2H_2_O and 0.5 g L^−1^ MgSO_4_/7H_2_O, and as a main carbon source, 15 g L^−1^ (dry weight) Avicel^®^ (Avicel PH-101, Sigma-Aldrich) for *L. arvalis*, or 15 g L^−1^ of wheat straw for *A. elegans* and *T. ljubarskyi*. Cultures were incubated in the dark at 30 °C with shaking at 120 rpm. The cultures were stopped 10 days after inoculation for *L. arvalis*, or 7 days for *A. elegans* and *T. ljubarskyi*, and the culture broths (secretomes) were filtered (using 0.2 μm polyethersulfone membrane, Millipore), diafiltered with 50 mM acetate solution buffer pH 5.2, concentrated (Pellicon^®^ 2 Ultrafiltration cassette with a 10 kDa cut-off polyethersulfone membrane, Millipore) and then stored at − 20 °C until use.

The total amount of proteins (Additional file 2: Table S1) was assessed using Bradford assays (Bio-Rad Protein Assay Dye Reagent Concentrate, Ivry, France) with a BSA standard that ranged from 0.2 to 1 mg mL^−1^.

### Proteomic analysis of secretomes

LC–MS/MS analysis of *L. arvalis*, *A. elegans*, *T. ljubarskyi* and *T. reesei* secretomes was performed as described [26]. Briefly, short SDS-PAGE runs (pre-casted Bis–Tris Mini Gels, Invitrogen, France) were performed, allowing 10 µg of proteins diafiltered from secretomes to migrate on 0.5 cm length. Each one-dimensional electrophoresis lane was cut into two slices of gel and protein identification was performed using PAPPSO “Plate-forme d’Analyse Protéomique de Paris Sud-Ouest” platform facilities. In-gel digestion was carried out according to a standard trypsinolysis protocol. Online analysis of peptides was performed with a Q-exactive mass spectrometer (Thermo Fisher Scientific, USA), using a nanoelectrospray ion source. Protein identification was performed by querying MS/MS data against the corresponding genome available at the Joint Genome Institute [32] for *A. elegans*, *T. ljubarskyi* and *T. reesei* strains, and against the transcriptome of *L. arvalis* (BioProject Accession: PRJNA244907), together with an in-house contaminant database, using the X!Tandem software (X!Tandem Cyclone, Jouy en Josas, France). All peptides matched with an E value lower than 0.05 were parsed with X!Tandem pipeline software. Proteins identified with at least two unique peptides and a log (E value) lower than − 2.6 were validated.

### Sequential hydrolysis of the substrates

**Fig. 1:**
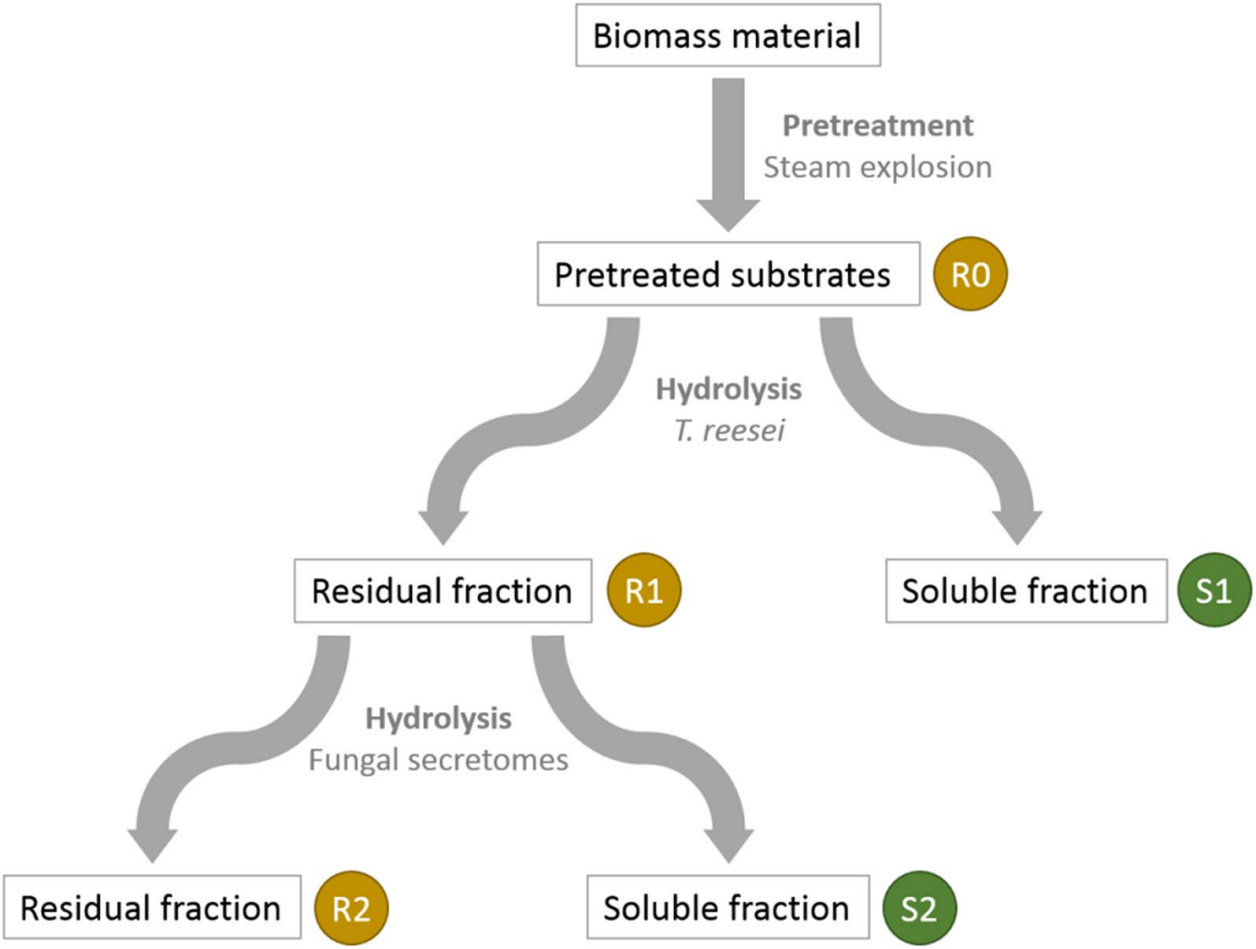
Biomass pre-treatment and sequential hydrolysis steps by fungal enzymatic secretomes of the biomass materials (poplar, wheat straw and miscanthus). R and S indicate residual (solid) and soluble fractions, respectively

Hydrolyses of the pre-treated R0 substrates were performed in a 20 L reactor using the secretome of *T. reesei*. Reactions were conducted with the washed and dried R0 substrates at a matter content of 10% (w/w) and an enzyme concentration of 15 mg g^−1^ substrate at 45 °C during 72 h in a sodium acetate buffer (50 mM, pH 4.8). The hydrolysis reactions were monitored by measuring the glucose released in the medium using the glucose oxidase assay (GM10, Analox). Hydrolyses from the remaining solids were washed with water and dried at 50 °C, yielding the R1 residues. The corresponding soluble liquid fractions were isolated by centrifugation and filtration (using 0.45 μm glass fibre membrane, GF/F, Whatman) were named S1 fractions (Fig. 1).

Each solid R1 residue was subsequently hydrolysed using each fungal secretome so that new S2 and R2 fractions were recovered (Fig. 1). Briefly, each R1 substrate (≈ 150 mg) was hydrolysed with either *L. arvalis*, *A. elegans T. ljubarskyi* or *T. reesei* secretomes, with 15 mg of protein g^−1^ of R1 sample at 2.5% (w/v) of dry matter in 0.02% (w/v) merthiolate. The enzymatic reaction was performed at pH 4.8 and 40 °C for all secretomes. These enzymatic conditions are usually suitable for most fungal secretomes but might not be optimal for these secretomes. After 72 h with shaking at 100 rpm, soluble fractions S2 were isolated by centrifugation and filtration (using 0.7 μm glass fibre membrane, GF/F, Whatman) and then stored at − 20 °C. Solid residual fraction R2 were washed twofold with 400 mL H_2_O and finally dried 96 h at 45 °C. Overall, steam-exploded (R0) substrates provided three R1 and S1 samples, and twelve R2 and S2 samples.

### Compositional analysis

The composition of the substrates was determined by the combination of different techniques. For the residual (solid) fractions, all the analyses were performed in triplicate on dry samples. Monosaccharides (including arabinose, xylose, glucose) were identified and quantified by gas–liquid chromatography after pre-hydrolysis and hydrolysis in sulphuric acid 13 M and 1 M, respectively [33]. Each sample was analysed after the first and second acid hydrolysis steps and after the second hydrolysis step only. The difference between the two results in glucose recovery was attributed to cellulose. Sugars were reduced to alditols with sodium borohydrure under agitation. Then alditols were acetylated using acetic anhydride and imidazole [34]. Alditol acetates were recovered in dichloromethane and analysed on Trace GC Ultra chromatograph (Thermo Scientific, USA) equipped with a TG-225MS column (Thermo Scientific, USA). Inositol was used as the internal standard and a standard solution of sugars was used for calibration.

Ester-linked phenolic acid content was determined after saponification in sodium hydroxide 2 M, addition of 3,4,5-trimethoxy-*trans*-cinnamic acid as the internal standard and extraction with ethylic ether [35]. The samples were analysed by HPLC on a C18 column (Vision HT, GRACE, Epernon, France) mounted on an Ultimate 3000 system (Thermo Scientific, Courtaboeuf, France) and eluted with a gradient of acetonitrile and acetate buffer pH 4.6.

Lignin content was quantified using a spectrophotometric method after acetyl bromide dissolution of lignocellulose, as previously described [36].

The FTIR spectra were recorded from KBr pellets made from 2 mg of samples mixed with 120 mg of KBr. The spectra were collected in transmission mode between 4000 and 700 cm^−1^ at 2 cm^−1^ intervals (Bruker Vector 22 spectrometer, France). The infrared spectra resulted from the co-addition of 200 interferograms. All infrared spectra in the 2000–700 cm^−1^ region were baseline-corrected and unit vector normalized using the OPUS software (version 7). Principal component analyses were applied to the normalised spectra of R0, R1 and R2 residues on Unscrambler 10.1 software (CAMO, Oslo Norway).

### Analysis of oxidized and non-oxidized oligosaccharides

Mono-, oligo-saccharides and their corresponding aldonic acids present in S2 were analysed by ionic chromatography (HPAEC) as already described [37], using non-oxidized oligosaccharides (Megazyme, Bray, Ireland) as standards. Gluconic acid (Megazyme) and corresponding C1-oxidized standards (from DP2 to DP6) were produced from non-oxidized cello-oligosaccharides using a cellobiose dehydrogenase treatment [37].

### Structural analysis of the substrates

Scanning electronic microscopy (SEM) was performed directly onto solid R samples with no metallisation, as previously described [25].

The particle size in R samples was determined by the Morfi instrument [38]. 100 mg of each sample was added in a 1 L beaker, then 2 mL of 95% ethanol were added before pouring 1 L of water. After mixing for 2 min at 120 rpm, the particles in suspension were analysed by the Morfi during 2 min so that the particles size distribution was obtained.

Sorption of the samples was performed by the Dynamic Vapour Sorption (DVS) method as previously detailed [39]. Sorption isotherms presenting water uptake versus humidity from 10 to 90% were measured in duplicate for 4–6 mg sample, in a temperature-controlled chamber maintained at 20 °C. Water uptake at 90% humidity for each sample was determined.

Solid-state ^13^C CP-MAS NMR was used to quantify the apparent cellulose crystallinity. The powder samples were packed into a zirconium specimen rotor and measured using a Bruker Avance spectrometer (^13^C frequency of 100 MHz) under ^13^C Cross-Polarization/Magic Angle Spinning (CP/MAS) with a cross-polarization contact time of 2 ms, a spinning rate of 12 kHz and an acquisition time of 35 ms. The recycle delay was 2 s for the CP/MAS measurements. The spectra were divided into spectral regions corresponding to different functional groups crystalline cellulose (86–94 ppm) and disordered cellulose (80–86 ppm). The integrated signal intensities were considered as proportional to the number of carbon atoms belonging to the corresponding to the functional groups. Apparent cellulose crystallinity was expressed as a percentage of the ratio of the integral of the C4 signal related to the crystalline contribution divided by the sum of the integrals of the C4 signal of both crystalline and disordered cellulose.

### Correlation analysis

Pearson’s coefficients were calculated between all possible pairs of markers and were displayed by using R software (R Foundation for Statistical Computing, Vienna, Austria) and *corrplot* package.

## Results and discussion

### Characterisation of raw lignocellulosic biomass samples

**Fig. 2:**
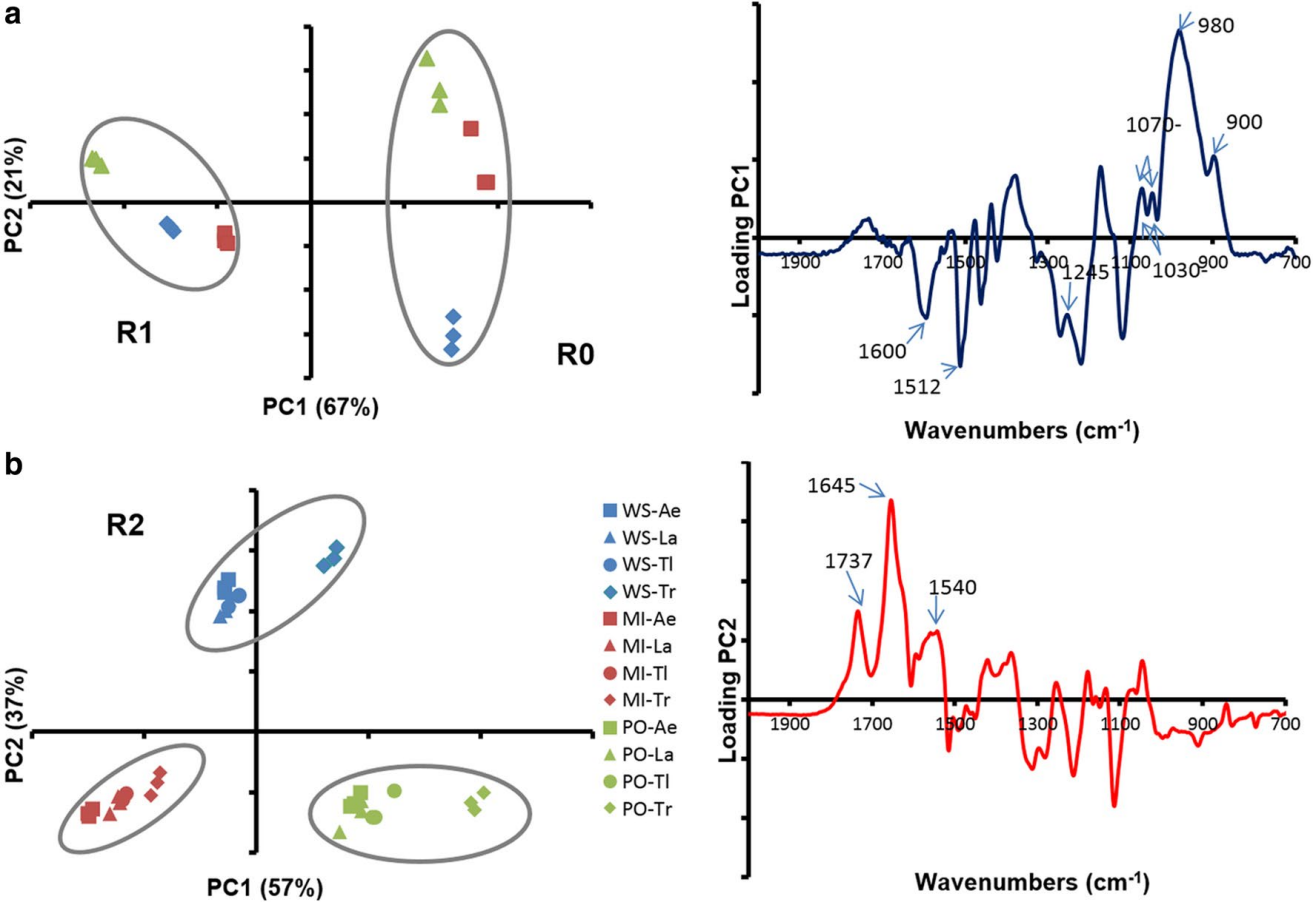
PCA of the biomass samples FT-IR spectra. A: R0 and R1, B: R2. Wheat straw (WS), blue; miscanthus (MI), red; poplar (PO), green; Ae, *A. elegans*; La, *L. arvalis*; Tl, *T. ljubarskyi*; Tr, *T. reesei*. Ellipses were drawn manually to highlight clusters

Wheat straw, miscanthus and poplar samples were selected as raw materials due to their relevance as industrial LB feedstocks. They were subjected to pre-treatment and different enzymatic hydrolysis steps described in Fig. 1. The steam explosion pre-treatment [25, 40] led to R0 samples. FT-IR spectra measurements followed by a principal component analysis (PCA) of the spectra showed that R0 from wheat straw was separated from the two other R0 samples (Fig. 2). Samples separated according to their content in sugar and phenolic compounds: miscanthus and poplar presented a higher content in phenolics (bands at 1512 and 1600 cm^−1^; Fig. 2) while wheat straw presented a higher content in hemicelluloses (bands at 1070 and 1045 cm^−1^; not shown). This was confirmed by the analysis of their chemical composition (contents in neutral sugars, lignin, phenolic acids, Table 1). All three biomass R0 samples displayed a high content in cellulose and lignin. As already reported in other studies [40–44], wheat straw R0 was the richest in xylose while miscanthus R0 was the richest in *p*-coumaric acid. These three LBs are chemically-contrasted substrates with potential various degrees of recalcitrance offering the possibility to assess fungal enzymatic secretomes.

**Table 1:** Composition of residual (solid) fractions R0, R1 and R2

Samples	Biomass	Secretome	Glc	Xyl	Ara	Lignin	Ferulic acid^a^	*p*-Coumaric acid^a^
R0	Wheat straw	None	54.7 ± 1.9	5.67 ± 0.23	0.39 ± 0.04	30.34 ± 0.07	0.06	0.13
	Miscanthus		59.4 ± 1.1	2.09 ± 0.04	0.39 ± 0.04	34.09 ± 0.92	0.07	0.55
	Poplar		53.3 ± 0.8	0.63 ± 0.01	0.11 ± 0.01	45.82 ± 1.74	0.01	0.01
R1	Wheat straw	*T. reesei*	26.8 ± 1.1	4.02 ± 0.17	0.59 ± 0.07	54.56 ± 0.58	0.08	0.26
	Miscanthus		36.6 ± 0.9	1.94 ± 0.05	0.51 ± 0.05	59.74 ± 0.21	0.07	0.89
	Poplar		20.7 ± 0.9	0.44 ± 0.02	0.12 ± 0.08	73.03 ± 1.43	0.01	0.02
R2	Wheat straw	*T. reesei*	16.6 ± 0.3	2.96 ± 0.10	0.48 ± 0.01	61.85 ± 0.76	0.08	0.28
	Miscanthus		31.3 ± 1.1	1.72 ± 0.08	0.49 ± 0.03	65.83 ± 1.04	0.07	0.99
	Poplar		13.8 ± 0.3	0.46 ± 0.04	0.07 ± 0.02	80.32 ± 4.51	0.01	0.02
	Wheat straw	*L. arvalis*	22.5 ± 0.6	3.64 ± 0.01	0.47 ± 0.04	54.85 ± 2.50	0.08	0.28
	Miscanthus		33.8 ± 1.3	1.91 ± 0.09	0.43 ± 0.04	53.47 ± 2.62	0.08	1.09
	Poplar		21.0 ± 0.2	0.63 ± 0.02	0.07 ± 0.01	74.89 ± 3.44	0.01	0.02
	Wheat straw	*A. elegans*	23.2 ± 0.3	3.68 ± 0.01	0.48 ± 0.01	48.39 ± 0.35	0.06	0.25
	Miscanthus		37.1 ± 0.7	2.05 ± 0.05	0.51 ± 0.03	59.18 ± 3.79	0.06	0.90
	Poplar		20.9 ± 0.2	0.60 ± 0.01	0.11 ± 0.01	67.62 ± 1.17	0.01	0.02
	Wheat straw	*T. ljubarskyi*	22.7 ± 0.8	3.63 ± 0.17	0.50 ± 0.04	48.33 ± 1.86	0.06	0.25
	Miscanthus		33.7 ± 1.1	2.00 ± 0.07	0.56 ± 0.03	61.70 ± 1.95	0.06	0.98
	Poplar		19.9 ± 0.5	0.65 ± 0.03	0.13 ± 0.03	76.11 ± 2.97	0.01	0.02

Results are expressed as % (w/w) of the residues. Values are means of independent triplicate measures

^a^Standard deviations were below 2%

### Compositional analyses of the fungal secretomes

**Fig. 3:**
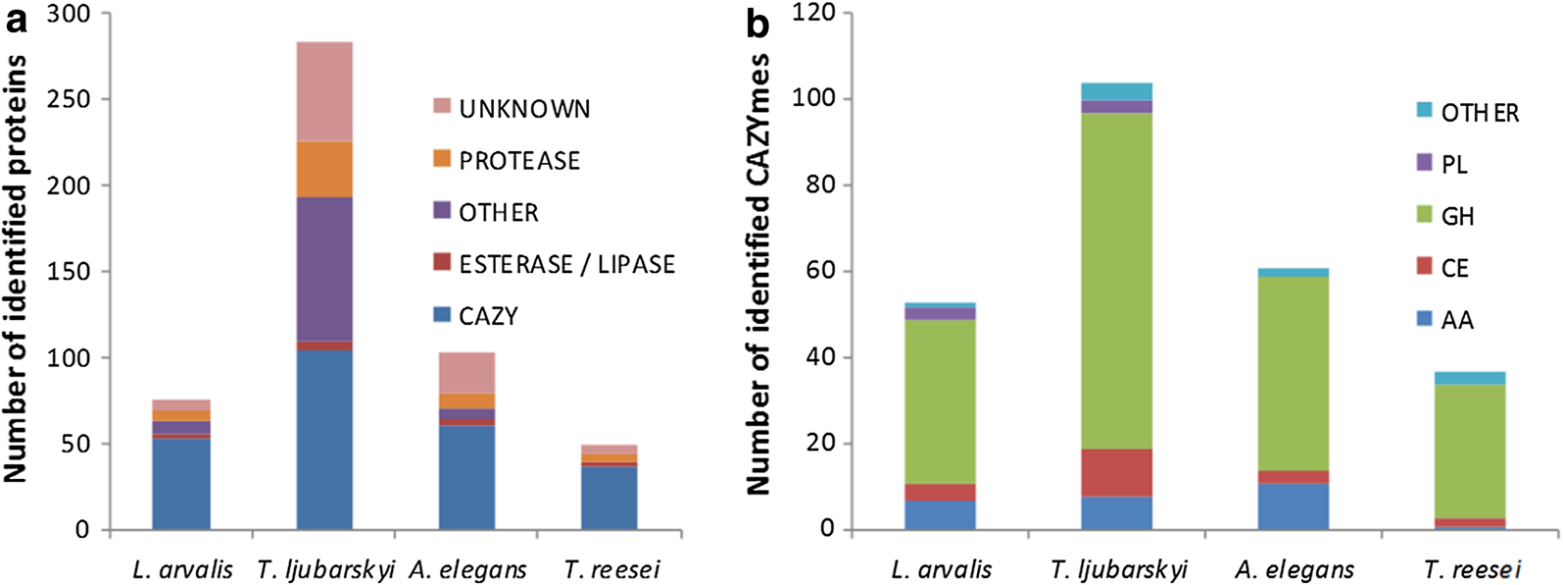
Distribution profile of **a** proteins and **b** CAZymes identified in fungal secretomes. AA, auxiliary activity enzyme; CE, carbohydrate esterase; GH, glycoside hydrolase; PL, polysaccharide lyase

The strains *L. arvalis*, *A. elegans* and *T. ljubarskyi* were selected for their ability to efficiently degrade LB based on previous studies [14, 26]. To determine the composition of the fungal secretomes and of the industrial *T. reesei* enzymatic cocktail, liquid chromatography–tandem mass spectrometry (LC–MS/MS) was performed and the data were analysed using mass matching against predicted proteins inferred from genomic and transcriptomic sequence data. Overall, 283, 103, 76 and 49 proteins were identified for *T. ljubarskyi*, *A. elegans*, *L. arvalis* secretomes and *T. reesei* enzymatic cocktail, respectively, for a total of 511 proteins (Fig. 3a). As expected, the highest proportion of carbohydrate-active enzymes (CAZymes) in the secretomes was found in that of *T. reesei* (more than 75%), followed by *L. arvalis* (70%) and *A. elegans* (59%), and the lowest was for *T. ljubarskyi* (less than 37%). Interestingly, the secretome of *T. ljubarskyi* contained a large number of proteins of unknown function. Among CAZymes, *T. ljubarskyi* had the highest number of glycoside hydrolases, while *A. elegans* had the largest number of auxiliary activity (AA) enzymes (Fig. 3b). More details of the CAZyme distribution in each secretome compared to *T. reesei* can be found in Additional file 3: Figure S2. It can be emphasized that the secretomes from the three basidiomycete strains (*L. arvalis*, *A. elegans* and *T. ljubarskyi*) display a very diverse set of CAZymes compared to the engineered strain of the ascomycete *T. reesei*, which lacks AA enzyme families that may target lignin components (AA1 laccases, AA2 peroxidases, AA3 oxidoreductases and AA5 glyoxal oxidases). Although the *T. reesei* secretome displays a diverse set of cellulose-acting enzymes with GH5, GH6 and GH12 endoglucanases and GH7 cellobiohydrolases, it lacks the GH131 endoglucanase abundantly secreted by the three basidiomycete fungi. The only secretome lacking cellulose-acting AA9 lytic polysaccharide monooxygenases (LPMOs) is the one of *L. arvalis* but it may compensate this deficit with the high level secretion of GH5_5 endoglucanases and GH7 cellobiohydrolases.

### Enzymatic degradation of the pretreated biomass samples

Steam-exploded biomass R0 substrates were subjected to two sequential hydrolyses (Fig. 1): a first hydrolysis by the *T. reesei* secretome led to residual (solid) samples R1 and soluble samples S1; then a second hydrolysis applied to R1 samples by each of the four different secretomes (*L. arvalis*, *A. elegans*, *T. ljubarskyi* and *T. reesei*) yielded residual samples R2 and soluble fractions S2.

The first hydrolysis kinetics by the *T. reesei* secretome on the three pretreated biomass substrates were followed during 72 h (Additional file 4: Figure S3), they were very similar regarding the initial velocity and hydrolysis evolution over 72 h. Wheat straw and poplar were hydrolysed more efficiently than miscanthus by the *T. reesei* secretome, as 77% of the initial cellulose content was released after 72 h for the two former substrates, against only 64% for miscanthus (Table 2). This is in agreement with the strongest known recalcitrance of miscanthus [44–47].

**Table 2:** Remaining glucose content in the residual (solid) products after the first and second hydrolysis and after both hydrolyses

	Secretome	Wheat straw (%)	Miscanthus (%)	Poplar (%)
First hydrolysis (R0 into R1)	*T. reesei*	22.7	35.6	22.9
Second hydrolysis (R1 into R2)	*T. reesei*	52.5	74.4	58.2
	*L. arvalis*	82.2	90.7	100.0
	*A. elegans*	79.4	92.5	94.5
	*T. ljubarskyi*	82.0	87.2	91.5
Both hydrolyses (R0 into R2)	*T. reesei*	11.9	26.5	13.3
	*L. arvalis*	18.7	32.3	22.9
	*A. elegans*	18.0	32.9	21.6
	*T. ljubarskyi*	18.6	31.0	20.9

Glucose content is expressed as the % of the initial glucose content either in R0 (considering first hydrolysis and both hydrolyses) or in R1 (considering second hydrolysis). Standard deviations for all measurements are below 5%

Composition of all solid fractions R0, R1 and R2 were analysed (Table 1), in order to determine for each hydrolysis step and for the overall hydrolysis steps the remaining glucose content in the residues (Table 2). After the first hydrolysis, the highest glucose content was measured in miscanthus (ca. 36% of R0 in R1) and was lower for wheat straw and poplar (ca. 23% each) (Table 2). After the second hydrolysis with each of the four different secretomes, *T. reesei* led to the best hydrolysis (remaining glucose from 52 to 74%), wheat straw being better hydrolysed than poplar and miscanthus. For the three other secretomes, the remaining glucose content in R2 in wheat straw was around 80% of R1, 90% for miscanthus and more than 90% in poplar, the latter appearing highly recalcitrant to the second hydrolysis. Regarding secretomes efficiency, only *T. reesei* and *T. ljubarskyi* were able to release glucose from all R1 samples.

Considering now the impact of the two sequential hydrolyses (Table 2), the most efficient secretome was that the *T. reesei,* with only 12 to 27% remaining glucose in the residual fractions R2, whereas the three other secretomes were close to each other with remaining glucose content spanning from 18% (for wheat straw) to 32% (for miscanthus), confirming the recalcitrance of LB in the order miscanthus > poplar > wheat straw. Since the *T. reesei* enzyme secretome used for the first and the second hydrolysis was identical, the highest glucose content in the residual fractions R2 after the second hydrolysis step in comparison to the residual fractions R1 after the first hydrolysis indicated that cellulose was much more prone to hydrolysis in the R0 samples than in the R1 residues. Consequently, the release of glucose from R0 samples conferred an increased recalcitrance to the R1 samples. Overall, large differences in glucose release were observed, depending both on the biomass species and the secretome used for the second hydrolysis. This difference could be due to the relative abundance and specificity of endoglucanases and cellobiohydrolases targeting amorphous and/or crystalline cellulose regions. In order to analyse the impact of the different secretomes in more details, chemical and structural characterization of the residual samples were performed.

### Substrate evolution during the first hydrolysis

The FTIR analysis (Fig. 2) showed that the R1 spectra differed from the R0 ones mainly by the higher intensity of the bands in the lignin region (1512 and 1600 cm^−1^, see loading PC1 Fig. 2a) following the disappearance of sugars induced by *T. reesei* secretome. Evolution in monosaccharide content (Table 1) indicates that while glucose content was decreased after hydrolysis, hemicellulose content was not altered, leading to an enrichment in phenolic compounds, in accordance with the FTIR data.

Properties of remaining cellulose were investigated by measuring its apparent crystallinity by NMR (Table 3). The steam explosion pretreatment leading to R0 samples is recognized as having a moderate effect on cellulose crystallinity [48]. Interestingly, apparent crystallinity after hydrolysis was not modified for wheat straw and miscanthus, and slightly decreased (from 54 to 51%) for poplar. This means that after the first hydrolysis, the nature of the remaining cellulose in R1 samples was not so different from the one of the R0 samples, validating that the *T. reesei* secretome used can degrade both crystalline and amorphous cellulose [49].

**Table 3:** Structural properties of the R0, R1 and R2 samples

Substrate	Biomass	Secretome	Crystallinity	Water sorption	Particle size
R0	Wheat straw	None	43	16.96	53.2
	Miscanthus		47	16.23	60.7
	Poplar		54	14.74	63.6
R1	Wheat straw	*T. reesei*	43	14.08	71.1
	Miscanthus		47	14.15	72.3
	Poplar		51	11.59	80.3
R2	Wheat straw	*T. reesei*	41	15.04	72.2
	Miscanthus		47	14.95	71.2
	Poplar		45	11.02	78.0
	Wheat straw	*L. arvalis*	41	15.29	71.5
	Miscanthus		47	14.46	66.1
	Poplar		50	12.23	80.9
	Wheat straw	*A. elegans*	41	16.15	72.3
	Miscanthus		49	15.36	69.2
	Poplar		49	12.35	80.4
	Wheat straw	*T. ljubarskyi*	37	15.58	70.5
	Miscanthus		46	15.28	73.5
	Poplar		50	12.12	80.3

Crystallinity is the apparent cellulose crystallinity expressed as a %. Water sorption indicates the water uptake % of the sample when humidity is 90%. Particle size indicates the % of particles whose size is equal or below 40 µm. Standard deviations for all measurements are below 5%

Water sorption properties were also analysed (Table 3) since they can reflect the chemical and structural organisation of biopolymers that cause their hydrophilic behaviour. Water sorption was considered in high relative air humidity (90%) in order to better discriminate samples, and even in such a humid environment, sorption by R0 samples was below 17%, indicating that samples were highly hydrophobic. After hydrolysis, sorption by R1 samples was even lower (14% or less). For R0 and R1 samples, water uptake was minimal for poplar samples, higher for miscanthus and wheat straw. These results were consistent with the increase in hydrophobic materials content such as lignin in R1 samples in comparison to R0 samples (Table 1).

**Fig. 4:**
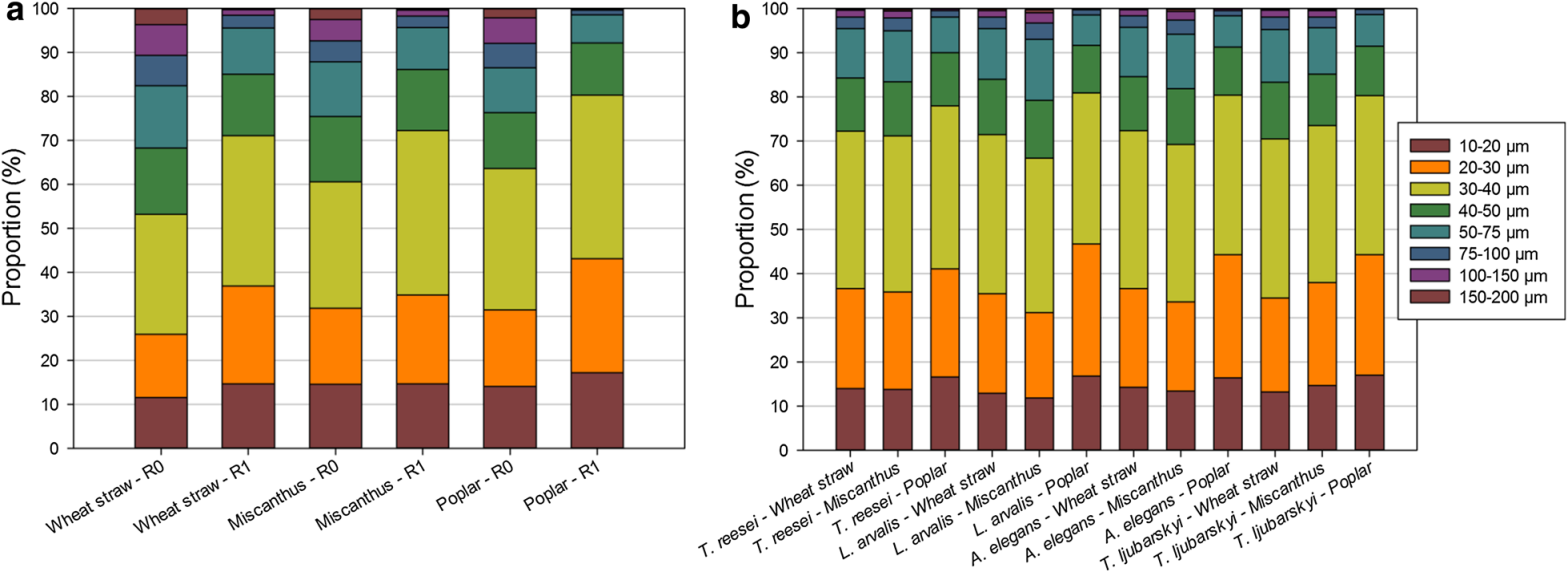
Distribution of particle sizes in **a** R0, R1 samples and **b** R2 samples

Regarding the particle sizes observed by SEM (Additional file 1: Figure S1), R0 samples from poplar appeared more uniform than those from miscanthus and wheat straw in which cell wall structures were still present. After the first hydrolysis, particle size in R1 samples seemed to be largely decreased for wheat straw and poplar, whereas miscanthus particles were more heterogeneous. This observation was in agreement with hydrolysis efficiency, which was higher for poplar and wheat straw than for miscanthus, indicating a potential relationship between particle size and hydrolysis efficiency, a trend previously described for wheat straw [50]. To further test this trend, quantitative analysis of particle size was done. Distribution of the particle sizes (Fig. 4a) showed that particles below 100 µm represented at least 90% of the population in R0 samples, and that even smaller particles below 40 µm were around 60%. After the first hydrolysis, the distribution was modified so that particles below 40 µm represented more than 70–80% of the total particles, the poplar samples distribution being the most modified by hydrolysis regarding their size. Since 40 µm seemed to be a relevant threshold to compare the evolution of particle sizes, the proportion of particles below 40 µm was gathered in Table 3.

### Substrate evolution during the second hydrolysis

FTIR spectra were acquired for all R2 residues. PCA (Fig. 2) showed that the biomass species had a stronger influence on spectra distribution than the secretome type, as indicated by the three groups corresponding to wheat straw, miscanthus and poplar. Inside each biomass group, spectra from R2 produced by *T. reesei* were separated from the other R2 samples produced by the three other secretomes. Discrimination according to the second component highlighted a higher content in protein in wheat straw (band at 1645 cm^−1^), other bands do not seem to be discriminant.

A detailed compositional analysis of each R2 was performed (Table 1). As previously mentioned, glucose content in the R2 samples were always lower or nearly identical to those of the R1 residues, *T. reesei* hydrolysis contributing to the highest decrease. Contents in hemicelluloses (followed thanks to xylose and arabinose) were only slightly affected in comparison to R1, whatever the biomass or secretomes considered. As a consequence, lignin relative content increased in all R2 residues, with the notable exception of hydrolysis by *A. elegans* on wheat straw (− 6.2 points) and poplar (− 5.5), *L. arvalis* on miscanthus (− 6.2) and *T. ljubarskyi* on wheat straw (− 6.2).

Apparent cellulose crystallinity decreased more after the second hydrolysis than after the first one, average index being close to 45% for R2 instead of 47% for R1 (Table 3). Even if these values are similar, there are some important differences between biomass types: miscanthus crystallinity did not evolve between R1 and R2 samples with an average difference of 0%, whereas this difference reached 3% and 6% for wheat straw and poplar, respectively.

Water sorption results (Table 3) did not highlight obvious differences between R1 and R2 samples, only confirming lower values for poplar than for miscanthus and wheat straw, and *T. reesei* R2 samples displaying decreased sorption. Finally, particle size analysis (Fig. 4b) indicated heterogeneous distribution of particles depending on biomass types and on secretomes, poplar having particle size distribution lower than miscanthus and wheat straw. Overall, the proportion of particles below 40 µm (Table 3) did not significantly decrease in R2 compared to R1, with the exception of miscanthus particles when hydrolysed by *L. arvalis* (− 6) and *A. elegans* (− 3) secretomes.

Overall, biochemical and structural results obtained after the hydrolysis by the secretomes highlighted different factors such as lignin content, water sorption, particle size and apparent cellulose crystallinity related to enzymatic hydrolysis efficiency. These factors can thus be considered as markers of sample recalcitrance. In order to better understand the relationships between these markers and how they can help predict hydrolysis, correlation analysis between these markers, together with other evaluated factors, was performed.

### Relationships between markers

**Fig. 5:**
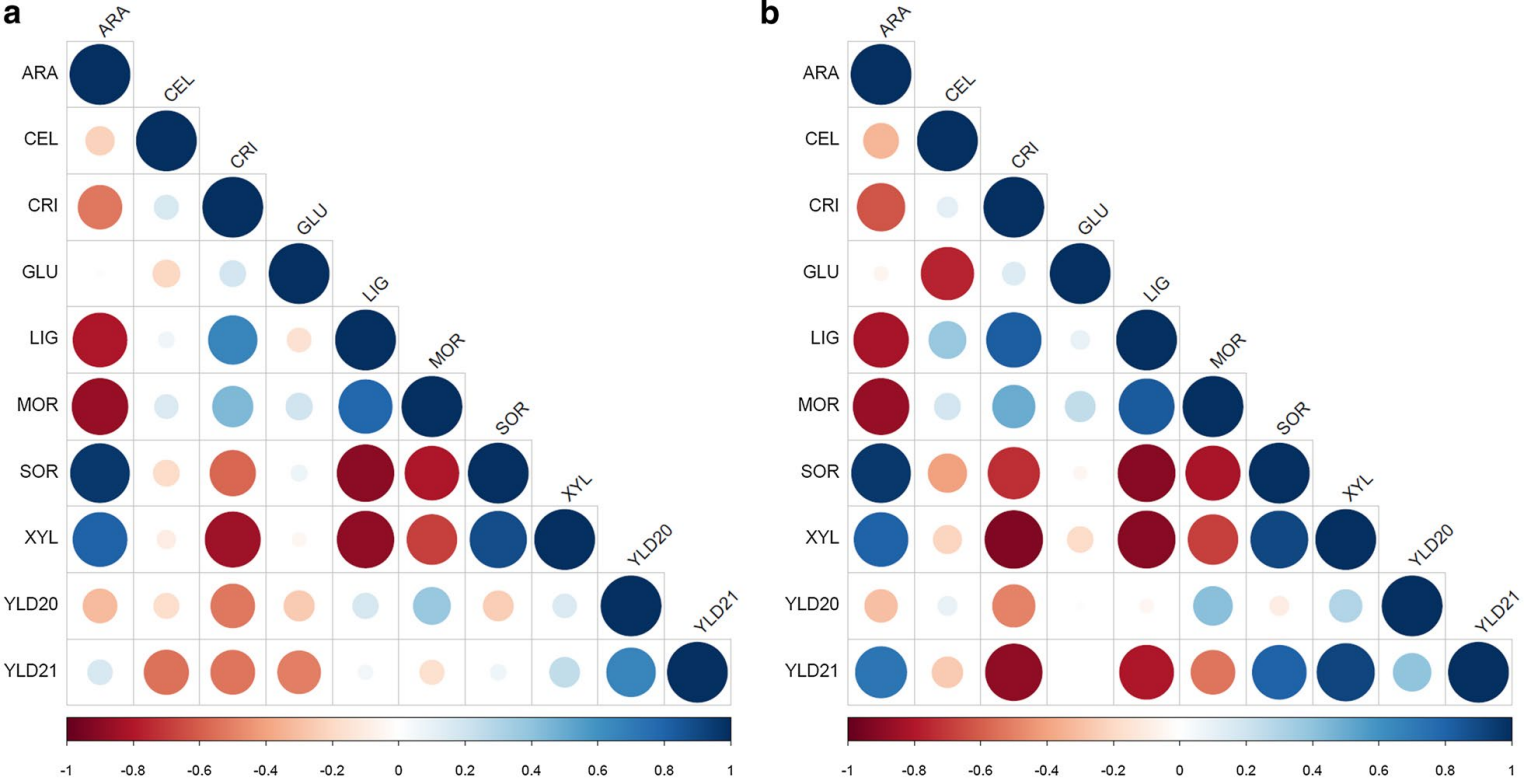
Correlation matrices between all measured markers for the R2 samples when considering **a** all secretomes and glucose yields R2 vs R0 and R2 vs R1; **b** all secretomes except that of *T. reesei* and glucose yields R2 vs R0 and R2 vs R1. Correlation intensity (as the Pearson’s coefficient) between two markers is reflected by the size of the disk and is positive when blue and negative when red. ARA, arabinose content; CEL, cellobionic acid content of the corresponding S2 fraction; CRI, apparent cellulose crystallinity; GLC, glucose content; GLU, gluconic acid content of corresponding S2 fraction; LIG, lignin content; MOR, % of particles of size is below 40 µm; SOR, % water uptake of sample at 90% water humidity; XYL, xylose content in R2; YLD20: glucose yield R2 vs R0; YLD21, glucose yield R2 vs R1

First, a correlation analysis was performed between the markers and the final glucose yield obtained between R2 and R0. When all secretomes are considered, the correlation matrix (Fig. 5a) indicated that the glucose yield was positively related to the proportion of particles of size below 40 µm (abbreviated as MOR; Pearson’s coefficient of + 0.6) and negatively correlated to arabinose content as ARA (− 0.6) and SOR (water sorption of sample at 90% air humidity) (− 0.55). Importantly, there was also strong correlation between (i) XYL (xylose content), ARA and SOR, (ii) LIG and MOR, and (iii) CRI (apparent cellulose crystallinity) and XYL. This means that the chemical composition of hemicelluloses was inversely related to lignin content and that it directly influenced the particle size together with apparent cellulose crystallinity. Water sorption appeared as a central marker: positively correlated with hemicellulose content, since hemicelluloses are rather hydrophilic; negatively correlated with lignin content and particle size, probably because small and hydrophobic particles are less prone to water interactions.

If the *T. reesei* secretome is not taken into account for the correlation analysis, based on the fact that its glycoside hydrolases content is substantially different from the three other secretomes (Fig. 3b), the matrix (Fig. 5b) remained similar to that including all secretomes (Fig. 5a), the glucose yield being not better correlated to other markers. This effect could be expected since the glucose yield calculated from R2 to R0 mainly originates from the first hydrolysis step. Also, regarding the content of oxidized sugars, a strong correlation was observed between cellobionic (CEL) and gluconic (GLU) acid contents (− 0.76), indicating that only the three secretomes from *L. arvalis, A. elegans* and *T. ljubarskyi* secrete sugar oxidases (mainly AA3 enzymes such as cellobiose dehydrogenases absent in *T. reesei*), as indicated by the analysis of their secretome in terms of CAZymes (Additional file 3: Figure S2).

In order to decipher the importance of these markers when considering recalcitrant substrates, the same correlation analysis were done considering the glucose yield between R2 and R1, which involved only one hydrolysis step with one secretome of the recalcitrant substrates previously hydrolysed by *T. reesei*. When considering all the secretomes (Fig. 5a), the same order in the correlations was observed as before (Fig. 5a). The most striking difference arose when the *T. reesei* secretome was removed from the correlative analysis (Fig. 5b). In addition to the still existing strong correlations previously described between the XYL, ARA, LIG, SOR, MOR, and CRI markers, glucose yield between R2 and R1 was highly correlated to apparent cellulose crystallinity (− 0.92), hemicellulose content (+ 0.63 for ARA and + 0.89 for XYL) and lignin content (− 0.76), water sorption (+ 0.70) and to a lesser extent to particle size (− 0.52).

The fact that more chemical and structural markers were highly correlated to the hydrolysis yield when the *T. reesei* secretome was not considered reflects its higher content in cellulose-acting enzymes (mainly GH5, GH6 and GH7) in comparison with other secretomes, which have proportionally more diverse CAZymes with a wider range of hydrolases and oxidases (Fig. 3b). More importantly, these results show that when considering recalcitrant substrates (pretreated and hydrolysed), some generic markers can help understanding the origin of hydrolysis limitations. Apparent cellulose crystallinity together with xylose and lignin contents appeared as strong markers of substrate recalcitrance. Although this was already largely described for raw or pretreated substrates [7], this was not clearly described for highly recalcitrant substrates such as those used in this study. Contrary, water sorption seems to be a pivotal marker since it reflects indirectly the chemical properties of both hemicellulose and lignin and their spatial arrangement. It is important to note that even if water sorption was already mentioned as a relevant predictor of enzymatic hydrolysis yield [22], our study expands the interest of such a marker to a wider set of biomass species (grasses and hardwood) and to highly recalcitrant substrates (hydrolysis residue) which are the key substrates to consider to make saccharification cost-effective. Also, since water sorption was also strongly correlated to the particle size, this reinforces the fact that reducing particle size is a key step to optimise hydrolysis [51]. Overall, among all the markers shown to be strongly correlated to enzymatic hydrolysis yield [47, 51–56], water sorption, even not the fastest one to measure, seems representative of the factors guiding hydrolysis.

## Conclusions

The two-step hydrolysis performed in this study by fungal secretomes led to a higher glucose yield than a one-step hydrolysis. This well-known phenomenon is mainly due to enzyme inactivation and inhibition during the course of hydrolysis [57], showing that enzyme non-specific interactions with hydrophobic features of the substrate are an essential behaviour to consider. Providing more diverse enzymatic activities (both hydrolytic and oxidative enzymes) does not necessarily help increasing glucose yield on highly recalcitrant substrates in comparison to a pure-hydrolytic secretome like that of *T. reesei*. However, the presence of oxidative enzymes in fungal secretomes can favour the release of oxidative sugars, some of them (e.g. gluconic acid) being considered as platform chemicals [58] or can help releasing lignin-derived molecules that serve as electron carriers used by LPMOs to increase the degradation of polysaccharides [59].

As expected, biomass recalcitrance mainly comes from cellulose crystallinity and lignin content, which increase during the course of hydrolysis, thus disfavouring hydrolysis mechanisms (through substrate inaccessibility and non-specific interactions of enzymes with lignin). Water sorption appears as a relevant marker representative of lignin and hemicellulose properties, and whose measurement can help to predict glucose yield, independently from biomass type and secretomes composition. To further investigate this observation, there is a need to better understand what happens at nanoscale over the course of lignocellulose degradation.

## Additional files

**Additional file 1: Figure S1.** SEM images of steam-exploded samples (R0, left) [25] and of residual solids fraction R1 from hydrolysed R0 samples (right, this study) for wheat straw, miscanthus and poplar (from top).

**Additional file 2: Table S1.** Production and protein content of fungal secretomes.

**Additional file 3: Figure S2.** Abundance of CAZymes and unknown proteins in the fungal secretomes. Abundances were determined based on the number of peptides unambiguously identified by LC–MS/MS. (a) Abundance of peptides for each CAZyme, represented by increasing shades of orange. (b) Abundance of proteins of unknown function grouped by orthology groups. Ortholog clustering was performed by OrthoDB [60]. The abundance of peptides corresponding to each orthology cluster is represented by increasing shades of green.

**Additional file 4: Figure S3.** Kinetic hydrolysis of R0 samples from wheat straw (square), poplar (circle) and miscanthus (triangle) by *T. reesei* secretome.

## Abbreviations

CAZyme: carbohydrate-active enzyme
LB: lignocellulosic biomass
LC–MS/MS: liquid chromatography–tandem mass spectrometry
PCA: principal component analysis
