# Supplementary material for: Tracking of enzymatic biomass deconstruction by fungal secretomes highlights markers of lignocellulose recalcitrance
Source: Biotechnol Biofuels. 2019 Apr 1;12:76. doi: 10.1186/s13068-019-1417-8 (PMC6442405; doi:10.1186/s13068-019-1417-8)
Supplement: Supplementary file 1 — Additional file 1: Figure S1. SEM images of steam-exploded samples (R0, left) [25] and of residual solids fraction R1 from hydrolysed R0 samples (right, this study) for wheat straw, miscanthus and poplar (from top). [file 13068_2019_1417_MOESM1_ESM.docx]

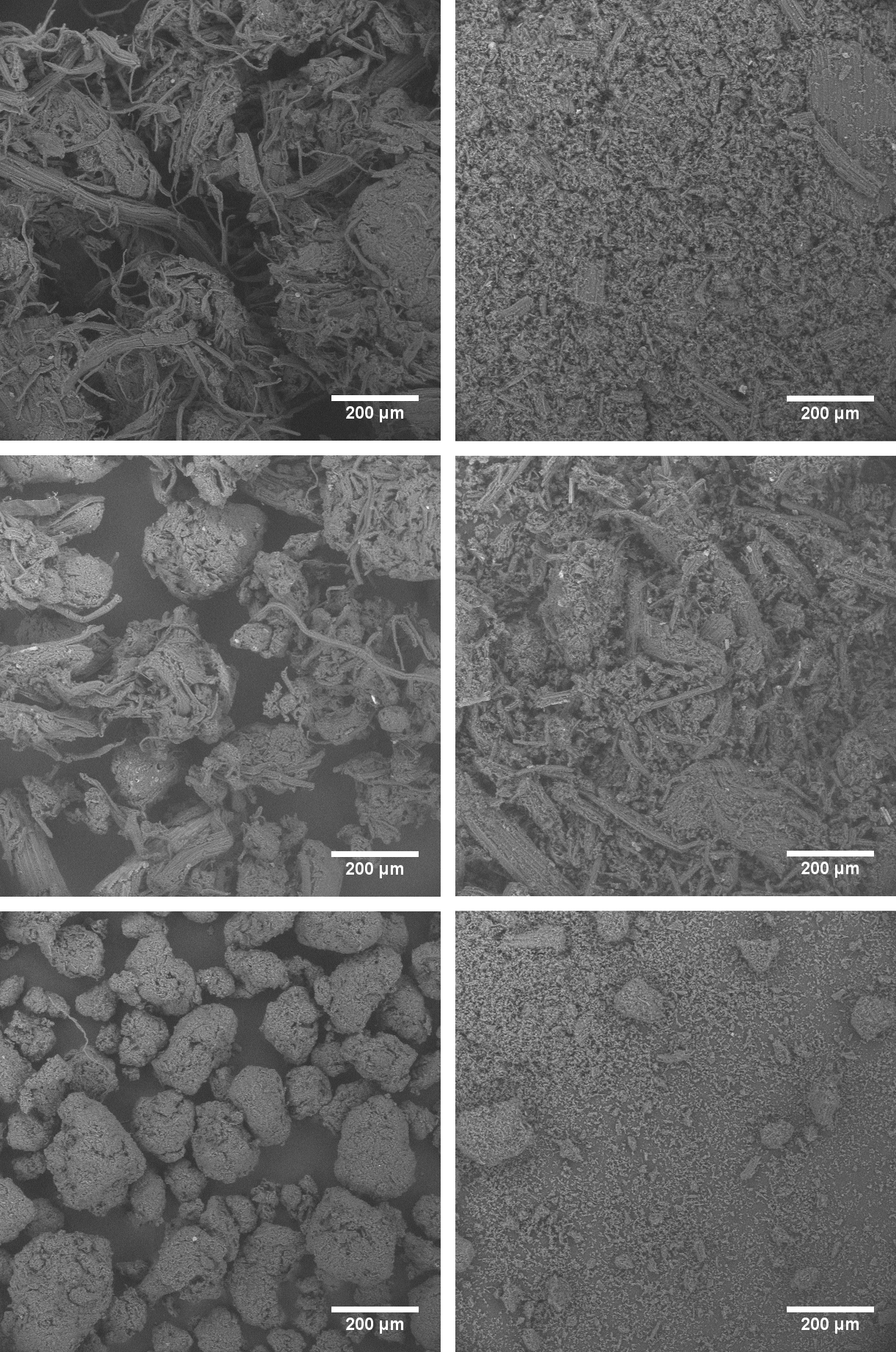


Figure S1. SEM images of steam-exploded samples (R0, left) {Auxenfans, 2017 #844} and of residual solids fraction R1 from hydrolysed R0 samples (right, this study) for wheat straw, miscanthus and poplar (from top).
