# Supplementary material for: Tracking of enzymatic biomass deconstruction by fungal secretomes highlights markers of lignocellulose recalcitrance
Source: Biotechnol Biofuels. 2019 Apr 1;12:76. doi: 10.1186/s13068-019-1417-8 (PMC6442405; doi:10.1186/s13068-019-1417-8)
Supplement: Supplementary file 2 — Additional file 2: Table S1. Production and protein content of fungal secretomes. [file 13068_2019_1417_MOESM2_ESM.docx]

| Strains | Inducer | Final volume (mL) | [protein] (mg.mL^-1^) |
| --- | --- | --- | --- |
| *L. arvalis* | Avicel | 290 | 1.7 |
| *A. elegans* | Wheat straw | 360 | 0.8 |
| *T. ljubarskyi* | Wheat straw | 400 | 1.1 |
| *T. reesei* |  |  | 46.9 |

Table S1. Production and protein content of fungal secretomes.
