# Supplementary material for: Tracking of enzymatic biomass deconstruction by fungal secretomes highlights markers of lignocellulose recalcitrance
Source: Biotechnol Biofuels. 2019 Apr 1;12:76. doi: 10.1186/s13068-019-1417-8 (PMC6442405; doi:10.1186/s13068-019-1417-8)
Supplement: Supplementary file 3 — Additional file 3: Figure S2. Abundance of CAZymes and unknown proteins in the fungal secretomes. Abundances were determined based on the number of peptides unambiguously identified by LC–MS/MS. (a) Abundance of peptides for each CAZyme, represented by increasing shades of orange. (b) Abundance of proteins of unknown function grouped by orthology groups. Ortholog clustering was performed by OrthoDB [60]. The abundance of peptides corresponding to each orthology cluster is represented by increasing shades of green. [file 13068_2019_1417_MOESM3_ESM.docx]

Figure S2. Abundance of CAZymes and unknown proteins in the fungal secretomes. Abundances were determined based on the number of peptides unambiguously identified by LC-MS/MS. (a) Abundance of peptides for each CAZyme, represented by increasing shades of orange. (b) Abundance of proteins of unknown function grouped by orthology groups. Ortholog clustering was performed by OrthoDB [58]. The abundance of peptides corresponding to each orthology cluster is represented by increasing shades of green.
