# Supplementary material for: Tracking of enzymatic biomass deconstruction by fungal secretomes highlights markers of lignocellulose recalcitrance
Source: Biotechnol Biofuels. 2019 Apr 1;12:76. doi: 10.1186/s13068-019-1417-8 (PMC6442405; doi:10.1186/s13068-019-1417-8)
Supplement: Supplementary file 4 — Additional file 4: Figure S3. Kinetic hydrolysis of R0 samples from wheat straw (square), poplar (circle) and miscanthus (triangle) by T. reesei secretome. [file 13068_2019_1417_MOESM4_ESM.docx]

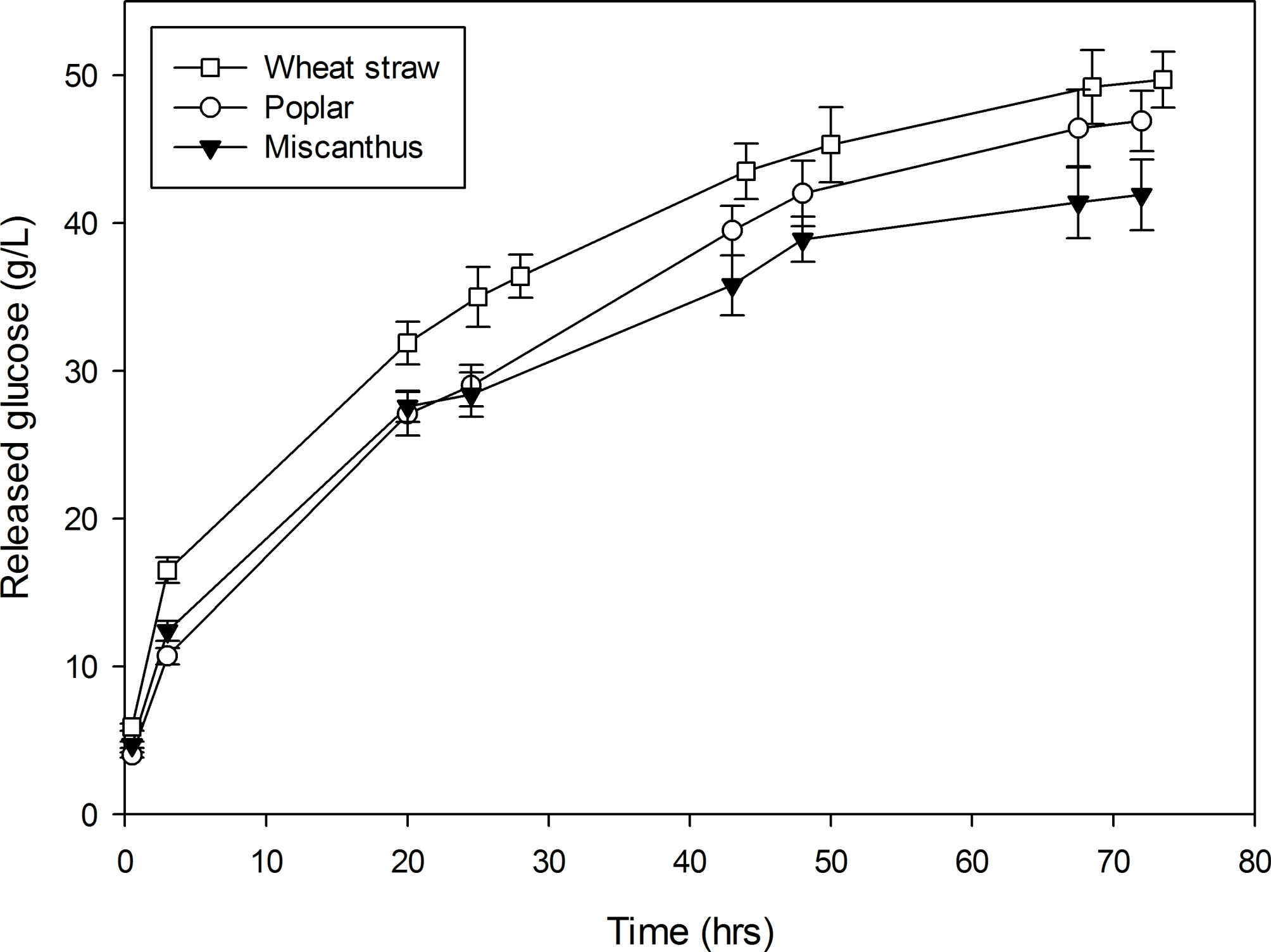


Figure S3. Kinetic hydrolysis of R0 samples from wheat straw (square), poplar (circle) and miscanthus (triangle) by *T. reesei* secretome.
